# Influencing factors of maternal online health information-seeking behavior

**DOI:** 10.3389/fpubh.2025.1592093

**Published:** 2025-07-04

**Authors:** Huajie Xu, Yanping Zhou, Xiaotong Wang

**Affiliations:** ^1^Department of Public Security Management, Shanxi Police College, Taiyuan, China; ^2^College of Economics and Management, Shandong University of Science and Technology, Qingdao, China; ^3^Institute of Higher Education Research, Shandong University of Science and Technology, Qingdao, China

**Keywords:** maternal, perceived usefulness, anxious emotion, social support, online health information-seeking behavior

## Abstract

**Introduction:**

As an important social group, mothers possess unique physiological and psychological characteristics. They may rely on Internet sources for obtaining health information about themselves and their children.

**Methods:**

Based on the Elaboration Likelihood Model (ELM) and Technology Acceptance Model (TAM), the influencing factors model of maternal online health information searching behavior is constructed. We collect 903 valid sample data through the questionnaire survey in China. Using SPSS software to empirically analyze the influence of different influencing factors on maternal online health information searching behavior.

**Results:**

Perceived usefulness mediated between the Internet information quality and online health information-seeking behavior, accounting for 59.46% of the variance. Anxiety mediated between maternal stress and online health information-seeking behavior, accounting for 19.40% of the variance.

**Conclusion:**

Internet information quality positively affected mothers’ perception of the usefulness of the information. Perceived usefulness and anxiety played a partial mediating role in the central and peripheral paths, respectively. Furthermore, social support effectively moderated the influence of maternal stress on their anxiety.

## 1 Introduction

The “Healthy China 2030” Plan emphasizes strengthening healthcare services for key populations and improving the health of women and children. On 31 May 2021, a policy allowing couples to have three children was implemented, leading to an increase in the number of mothers in China. Both society and several families support the “two children” and “three children” policies, which intensify social competition. Mothers not only experience family stress, including the stressors of managing family life, raising children, and supporting older adults, but also work-related stress, such as employment discrimination, challenges in self-development, conflicts related to maternity leave, and stress induced by their unique physiological characteristics. The problem of physical and psychological “sub-health” is becoming increasingly serious, which affects the health of mothers and their children. Therefore, studying maternal health information-seeking behaviors is of critical importance.

Health information refers to information about physical and mental health (diseases, healthcare, and health maintenance). Online health information-seeking behavior refers to the entire process in which network users search, obtain, identify, apply, provide feedback, and evaluate health information in a specific environment with the goal of performing a specific health information search task (1). Soroya et al. (2) observed that the media played a significant role in transforming the medical environment and the emergence of medical consumers. An increasing number of people are using the media to obtain health information, especially after the COVID-19 pandemic (3). With the rapid development of information technology in China, the number of Internet users in China has reached 1.092 billion as of December 2023. Many social media platforms offer health coaching to individuals lacking medical knowledge (4). In this context, mothers form a large group who seek online health information. The Internet is the primary means for young mothers to obtain information about themselves and their babies during pregnancy and after delivery. Traditional offline information sources, such as doctors, friends, and books, have become secondary (5). Search engines (Baidu, Yahoo, Google, etc.), social media (WeChat, Weibo, QQ, etc.), and medical health information websites (Mama.com, Dingxiang Doctor, 39Health.com, etc.) have become the primary sources for mothers to obtain health information. Additionally, owing to the characteristics of anonymity and timeliness of information on the Internet, mothers can safely search for relatively sensitive information (6) when they need to learn about themselves and their child’s health, including psychological distress, health during pregnancy, and postpartum (7).

Previous scholars have primarily focused on the online health information-seeking behavior of older adults (8), adolescents (9, 10), college students (11, 12), minorities (1), and urban and rural residents (13). Previous literature indicates that online health information-seeking behavior is not only related to users’ demographic characteristics, such as sex, race, residence, age, income, and education level (14), but also to their psychological needs, online health information quality, and perceived usefulness. However, a gap in the literature about the factors influencing maternal online health information-seeking behavior still exists. Therefore, considering the implementation of the “three children” policy in China, the improvement of women’s social status, and their unique physiological and psychological characteristics, it is necessary to determine the factors influencing their online health information-seeking behavior to improve the level of maternal and child health.

This study makes significant innovative contributions in both theoretical construction and methodological application. Primarily, we pioneered the integration of the Elaboration Likelihood Model (ELM) and Technology Acceptance Model (TAM), establishing, for the first time, a “dual-path multidimensional” analytical framework specifically for maternal health information behavior, which breaks through the explanatory limitations of traditional single-theory models. Methodologically, our groundbreaking integration of mediation and moderation analyses not only confirmed the dual mediating mechanisms of perceived usefulness and anxiety, but more importantly, were the first to identify the specific moderating role of social support in the stress-anxiety pathway. This novel discovery provides fresh perspectives for understanding the complex interactive mechanisms of maternal health information behavior in the digital era.

This study is divided into five sections. The first section provides a brief introduction. The second section includes the materials and methods, including the theoretical basis, literature review, and discussion of the methods. The third section presents our empirical results. The fourth section discusses the research results, their theoretical and practical significance, and limitations. The final section summarizes the research conclusions.

## 2 Materials and methods

### 2.1 Theoretical basis

#### 2.1.1 The Elaboration Likelihood Model (ELM)

**Figure 1:**
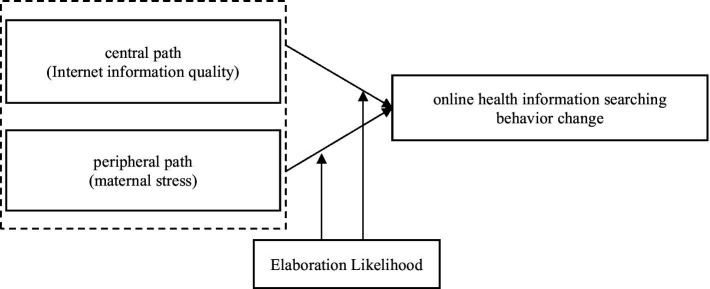
Core idea of the ELM.

ELM was first proposed by Petty and Cacioppo (15). Currently, this theoretical model is widely used in consumer information processing research and searching behaviors. It provides an overall framework for individuals to understand the dual-path (central and peripheral paths) influence process behind the information persuasion effect. On the one hand, under the influence of the central path, individuals make rational decisions through a series of tests and attempt to judge and evaluate information from a logical perspective. Ricco (16) and Zhou et al. (17) revealed that Internet information quality directly affects individuals’ perceptions of the usefulness, accuracy, and credibility of online information, which consequently determines their loyalty to a specific website. Filieri and McLeay (18) studied users’ online travel information adoption behaviors and found that the accuracy, relevance, and timeliness of the information had a significant positive impact on their information selection behavior. Therefore, among various factors, users’ perceptions of the accuracy, timeliness, and usefulness of the information are more suitable for constructing the central path of the factors influencing maternal online information-seeking behavior. On the other hand, when individuals are influenced by the peripheral path, they are often emotionally tied and have no motivation or ability to think rationally. Consequently, they can judge the credibility of the information only through simple peripheral cues and do not have in-depth rational cognition. Lagoe and Atkin (19) found that, compared to men, women experience higher anxious emotions, are more sensitive to health risk perceptions, and engage in more frequent health information-seeking behaviors based on emotional factors. Therefore, anxiety caused by individual stress is more suitable for constructing a peripheral path of the factors influencing maternal online health information-seeking behavior. The core concept of the ELM is shown in Figure 1. Therefore, a dual-path model was constructed to explore the factors influencing maternal online health information-seeking behavior using the ELM as the theoretical basis.

#### 2.1.2 The Technology Acceptance Model

TAM was proposed by Davis (20). This model refers to the influence of users’ utilization of information technology (IT) on their behavioral intentions. It is primarily used to explore the influence of external factors of information on users’ beliefs, attitudes, cognitions, and intentions, which then affect the use of information systems. Perceived usefulness is a value effect that refers to the fact that, when users use a particular information system, they subjectively believe that the information provided by it is valid and reliable and can influence their cognitive judgments. Davis (20) found that users’ cognition about the usefulness of information positively promoted their usage behavior. Many scholars, such as Korfiatis et al. (21), Liu et al. (22), Caffaro et al. (23), and Mican et al. (24), have used perceived usefulness to measure users’ recognition of information. Based on the true situation of this study, we explored the influence path of maternal online health information-seeking behavior. Therefore, we used the TAM as the theoretical basis to explore the effect of perceived usefulness as an intermediate variable on maternal online health information-seeking behavior.

### 2.2 Literature review

#### 2.2.1 The mediating role of perceived usefulness in the central path

With the advancement of Internet technology, online health information has gradually addressed the disadvantages of traditional methods of obtaining health information, such as the spatiotemporal and unequal distribution of information resources (25). At present, users can obtain health information more efficiently and in a timely manner. However, the effect of online health information on users’ behaviors is associated with their evaluation of online health information through their cognitive ability (26, 27). Only when mothers perceive online health information is accurate, credible, and useful—that is, they perceive its usefulness—do they use the information and continue with this behavior (28). Mothers’ perception of the usefulness of the information is influenced by the quality of Internet information and information sources (29, 30). Neither the quality of the information nor the quality of the information source has a direct impact on maternal online health information-seeking behavior; however, the former acts on their cognition and affects their behavior. Therefore, the present study defines perceived usefulness as whether the online health information is valuable to mothers and addresses health problems encountered during pregnancy, puerperium, and lactation.

Concerning the relationship between Internet information quality and perceived usefulness, and the relationships between information source quality and perceived usefulness, Jin et al. (31), Shen et al. (32), and Cheung et al. (33) believe that information quality and information source quality have a significant positive impact on users’ perceptions of information usefulness. Concerning the relationship between perceived usefulness and online information-seeking behavior, Li et al. (34) believe that how users choose and ultimately perform information-seeking behaviors primarily depends on their perception of the information’s usefulness and reliability and that perceived usefulness plays a positive role in promoting users’ information use attitudes and searching behavior intentions.

Thus, the present study introduced perceived usefulness as an intermediary factor in the central path research model of the factors influencing maternal online health information-seeking behavior. Therefore, the following hypothesis was proposed:

*H1*: Perceived usefulness mediates the relationship between Internet information quality (information quality and information source quality) and maternal online health information-seeking behavior.

#### 2.2.2 The mediating role of anxiety in marginal pathways

Housewife is no longer the only label or choice for married women, owing to the enhancement in women’s social status, and the employment rate of women has been increasing (35). Mothers assume multiple roles in this context. They not only experience family stress, including stressors of family life, raising children, and supporting older adults, but also experience work-related stress, including employment discrimination, self-development challenges, and conflicts surrounding maternity leave and position, coupled with their unique physiological characteristics. Together, it increases their physical and psychological stress (36, 37). When mothers perceive the threat of stress, they experience negative emotions that affect their physical and mental health (38). Studies have shown that, in addition to seeking support from family members, relatives, and friends, anxious individuals also seek comfort and solutions through online health information-seeking behaviors (39). Lagoe and Atkin (19) found that, compared to men, women experienced higher anxiety, greater sensitivity to perceived health risks, and were more likely to search for health information based on emotional factors more frequently. Through online health information searches, mothers can gain an initial understanding of their health problems and use the information gathered from the Internet to self-diagnose and develop health strategies. Additionally, the anonymity of the Internet enables mothers to eliminate their privacy concerns and ask relatively private questions about pregnancy and childbirth.

Therefore, work, family, and physiological stress were the primary sources of maternal stress. When stress exceeds mothers’ psychological capacity, they experience anxiety, which affects their online health information-seeking behavior. In the present study, anxiety was introduced as a mediating factor in the peripheral path research model of factors influencing maternal online health information-seeking behavior. Therefore, the following hypothesis was proposed:

*H2*: Anxiety plays a mediating role between maternal stress (work, family, and physiological stress) and maternal online health information-seeking behavior.

#### 2.2.3 The moderating effect of social support

With the three-fold stress of work, family, and physiology, along with fierce social competition, mothers often feel confused about their identity. Therefore, they require greater care, understanding, and external support. Social support refers to an individual’s perceptions of care, help, support, and recognition from family, friends, colleagues, or society (40). Ta'An et al. (41) found that the same stressful situation can have different effects on different individuals. People who received more support from family or friends had stronger psychological endurance and were physically and mentally healthier than those who rarely received similar support. Barrera and Ainlay (42) divided social support into six categories according to its function: (1) material aid, such as practical help with money and other materials; (2) behavioral assistance, such as work and sharing physical labor; (3) intimate interaction behavior, such as listening, showing respect, concern, and understanding; (4) guidance, such as providing help, information, and guidance; (5) feedback, such as providing personal feedback about their behaviors, thoughts, and feelings; and (6) positive social interaction, such as participating in entertainment and relaxing social interactions. Turner (43) reported that social support can effectively reduce the impact of anxiety on individuals’ physical and mental health and enhance their ability to manage stress.

Therefore, the present study introduced social support as a moderating factor within the peripheral pathway of the research model on factors influencing maternal online health information-seeking behavior. The following hypothesis was proposed:

*H3*: Social support negatively moderates the relationship between maternal stress (work, family, and physiological stress) and anxiety.

**Figure 2:**
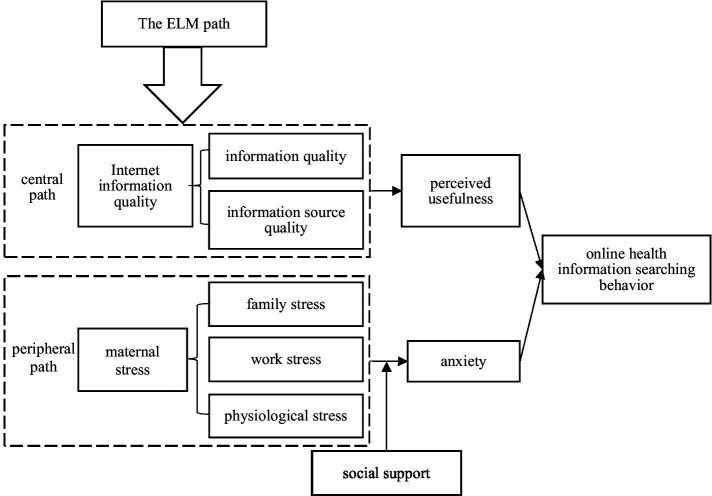
Research model framework.

An influencing factor model of maternal online health information-seeking behavior was constructed based on the above hypothesis, as shown in Figure 2.

### 2.3 Data and methods

#### 2.3.1 Data collection

This study utilized a questionnaire to explore the factors influencing maternal online health information-seeking behavior. The maturity scale in the existing literature was used to develop the questionnaire items. Our survey participants included mothers. Before the initiation of data collection, pilot tests were conducted to ensure that the measurements were clear and understandable by the participants. Based on the results of the pilot test, as well as the comments and feedback from the respondents, we modified certain descriptions and wording in the questionnaire to make it easier to understand, while preserving the original meaning.

The questionnaire comprises three sections, including the preface, basic demographic information, and main variable items. The basic demographic information included age, education, job, income, and birth experience. The questionnaire survey was administered using a combination of online and offline methods between 1 February 2022 and 31 May 2022. Mothers in China were invited to participate in the online survey through WeChat groups, QQ groups, and Weibo, while offline questionnaires were distributed to mothers who visited the Qingdao Maternal and Child Health Hospital during the survey period. After screening, questionnaires with a response time of less than 60 s, extreme responses, or a repetitive answering pattern were eliminated. Approximately 903 questionnaires, 521 valid online questionnaires, and 382 valid offline questionnaires were collected. Table 1 displays the participants’ characteristics.

**Table 1:** Basic demographic characteristics of the participants.

Variables	Options	Frequency	Proportion (%)
Age	18 ~ 23	135	14.95
	24 ~ 28	396	43.85
	29 ~ 34	285	31.56
	35 ~ 50	87	9.64
Job	Housewife	93	10.30
	Personnel of agencies and institutions	153	16.94
	Corporate employees	315	34.88
	Self-employed	96	10.63
	Freelance	181	20.05
	Unemployed	30	3.32
	Student	21	2.33
	Other	14	1.55
Education level	Junior high and below	76	8.42
	High school/technical secondary school	160	17.72
	Junior college	236	26.13
	Undergraduate	338	37.43
	Postgraduate and on	93	10.30
Monthly income (YUAN)	≤1,999	198	21.93
	2,000 ~ 3,999	303	33.55
	4,000 ~ 6,999	291	32.23
	7,000 ~ 9,999	70	7.75
	≥10,000	41	4.54
Birth experience	First childbirth	687	76.08
	Have childbirth experience	216	23.92

The present study examined six main variables: online health information-seeking behavior was the dependent variable, Internet information quality (central path) and maternal stress (peripheral path) were independent variables, perceived usefulness (central path) and anxiety (peripheral path) were mediating variables, and social support (peripheral path) was the moderating variable. All variables were measured on a 5-point Likert scale; responses included strongly agreed (5), relatively agreed (4), generally (3), relatively disagreed (2), and strongly disagreed (1). Table 2 shows the specific settings of the principal variables.

**Table 2:** Measurement index and reliability of the variables.

Variables			Cronbach’s alpha
Internet information quality (56)	Information quality	Online health information is updated on time	0.937
		Online health information is accurate	
		Online health information is comprehensive	
	Information source quality	Providers of information are reliable	
		Providers of information are professional	
		Platforms that provide health information are reliable	
Maternal stress (57)	Family	My family’s financial burden is heavy	0.908
		I often have conflicts with my family	
		Supporting older adults and raising children is a burden	
		Family and work cannot be taken care of at the same time	
	Work	My workload is heavy	
		I am thinking about work when I wake up	
		I am still thinking about work when I sleep at night	
	Physiological	I feel anxious and irritable during pregnancy	
		I feel irritable and tired during the puerperium	
		I feel depressed during breastfeeding	
Anxiety (58)	Stress makes me uncomfortable		0.904
	Stress makes me nervous		
	Stress makes me irritable		
Perceived usefulness (59)	Online information can provide me with professional health knowledge		0.950
	Online information can answer my health doubts		
	Online information can help me improve my health		
Social support (60)	Some people (family, friends) will accompany me when I need		0.964
	I will share my sadness with some people		
	My family can support me financially		
	My family can give me emotional support		
	Some people comfort me when I have difficulties		
	I can talk to my family about my problems		
	My family can rationally assist me in making decisions		
Online health information-seeking behavior (61)	I often search for health information		
	I spend a lot of time searching for health information		0.918
	I termly search for health information		

#### 2.3.2 Method

This study uses mediation and moderation effect models to empirically analyze the influencing factors of maternal online health information-seeking behavior. Mediation and moderation models, as core statistical methods in behavioral science research, possess distinctive analytical advantages and practical value in health information behavior studies. The mediation model can profoundly elucidate the underlying mechanisms between variables by identifying and quantifying mediators (such as perceived usefulness and anxiety identified in this study). This approach not only addresses how independent variables influence dependent variables but also decomposes the relative contributions of direct and indirect effects. The moderation model, on the other hand, effectively identifies the boundary conditions of influence relationships by examining how moderators (e.g., social support in this study) alter the strength or direction of the relationship between independent and dependent variables, thereby providing a basis for developing differentiated intervention strategies.

Before performing the regression analysis, the reliability and validity of the questionnaire data should be tested. Therefore, first, the reliability of the questionnaire was assessed. Cronbach’s coefficient values of each variable in Table 2 are greater than 0.9, and the overall Cronbach’s coefficient value of the questionnaire was 0.915, which is greater than 0.9. These results indicated the high reliability of the data obtained through the questionnaire. Second, the validity of the questionnaire was assessed. The results showed that the KMO value of the questionnaire was 0.9, which is greater than 0.8, indicating the validity of the questionnaire data. Bartlett’s sphericity test was 0.00 (which is less than 0.05), which indicated that the variables on the scale were significantly correlated. The cumulative variance explanation rate after the six-factor rotation was 76.464%, which is greater than 50%, indicating that the information on the questionnaire’s main variables could be extracted effectively. The reliability and validity of the questionnaire were relatively satisfactory and could be analyzed further.

## 3 Results

### 3.1 The mediating role of perceived usefulness in the central path

Models 1, 2, and 3 in Table 3 analyzed the mediating effect of perceived usefulness on the relationship between Internet information quality (information quality and information source quality) and maternal online health information-seeking behavior. Model 1 assessed the relationship between Internet information quality and online health information-seeking behavior. Model 2 assessed the relationship between Internet information quality and perceived usefulness. In Models 1 and 2, the obtained coefficients passed the 1% significance test. Model 3 introduced perceived usefulness in the relationship between Internet information quality and online health information-seeking behavior. The coefficient of the independent variable was *β* = 0.268, which was less than the original coefficient of 0.660, and the coefficient of the intermediary variable was *β* = 0.549. Both the independent and intermediary variables passed the significance test at the 1% level. These results revealed that, when controlling for the perceived usefulness of the intermediate variable, the correlation coefficient between the independent and dependent variables was significantly reduced. Perceived usefulness mediated the relationship between Internet information quality and online health information-seeking behavior, accounting for 59.46% of the variance, which indicates a partial mediation effect. These results supported Hypothesis 2. Therefore, mothers were concerned about the quality of online health information and information providers. The higher the quality of Internet information and information sources, the better the perceived usefulness of online health information among mothers, which, in turn, increases their online health information-seeking behavior.

**Table 3:** Mediating effect test of perceived usefulness in the central path.

	Model 1		Model 2		Model 3	
	Online health information searching behavior		Perceived usefulness		Online health information searching behavior	
	*β*	*t*	*β*	*t*	*β*	*t*
Constant	1.139^**^	6.822	1.098^**^	6.779	0.537^**^	3.606
Internet information quality	0.660^**^	14.967	0.715^**^	16.723	0.268^**^	5.582
Perceived usefulness					0.549^**^	13.079
Adjust *R^2^*	0.339		0.390		0.525	
*F*-value	224.016		279.673		241.430	

^*^ means *p* < 0.05, ^**^ means *p* < 0.01.

### 3.2 The mediating role of anxiety in the peripheral path

Models 1, 2, and 3 in Table 4 assessed the mediating effect of anxiety on the relationship between maternal stress (family, work, and physiological stress) and online health information-seeking behavior. Model 1 examined the relationship between maternal stress and online health information-seeking behavior. Model 2 examined the relationship between maternal stress and anxiety. In Models 1 and 2, the obtained coefficients passed the 1% significance test. Model 3 introduced anxiety into the relationship between maternal stress and online health information-seeking behavior. The coefficient of the independent variable was *β* = 0.281, which was less than the original coefficient of 0.349. The coefficient of the intermediate variable was *β* = 0.201. The intermediate variable passed the 1% significance test. The results revealed that the older the mother and the higher the level of education, the greater their perceived pressure and anxiety. Furthermore, when controlling for anxiety as the intermediate variable, the correlation coefficient between the independent and dependent variables was significantly reduced. Anxiety mediated the relationship between maternal stress and online health information-seeking behavior, accounting for 19.40% of the variance, which indicates a partial mediation effect. These results supported Hypothesis 2. Therefore, higher maternal stress produced greater anxiety, which, in turn, increased their online health information-seeking behavior.

**Table 4:** Mediating effect test of anxiety in the peripheral path.

	Model 1		Model 2		Model 3	
	Online health information-seeking behavior		Anxiety		Online health information-seeking behavior	
	*β*	*t*	*β*	*t*	*β*	*t*
Constant	2.378^**^	10.999	2.374^**^	11.985	1.901^**^	7.745
Maternal stress	0.349^**^	5.642	0.337^**^	5.948	0.281^**^	4.444
Anxiety					0.201^**^	3.896
Adjust *R^2^*	0.066		0.073		0.096	
*F*-value	31.836		35.376		24.027	

^*^ means *p* < 0.05, ^**^ means *p* < 0.01.

### 3.3 The moderating role of social support

Models 1, 2, and 3 in Table 5 assessed the moderating effect of social support on the relationship between maternal stress (family, work, and physiological stress) and anxiety. Model 1 examined the relationship between maternal stress and anxiety. Model 2 investigated the relationships between maternal stress, social support, and anxiety. Model 3 introduced the interaction terms between maternal stress and social support in the relationship between maternal stress, social support, and anxiety. Regarding the analysis of the moderating effect, the *F*-value increased from 3.928 to 19.392 from Model 2 to Model 3, indicating a significant improvement. The interaction between maternal stress and social support in Model 3 was significant at the 1% level. These results revealed that social support negatively regulated the relationship between maternal stress and anxiety. Thus, Hypothesis 3 was supported. Therefore, social support effectively regulates maternal stress. Mothers experienced less anxiety when they received more care and support from family and friends.

**Table 5:** Test of the moderating effect of social support.

	Model 1		Model 2		Model 3	
	Anxiety		Anxiety		Anxiety	
	*β*	*t*	*β*	*t*	*β*	*t*
Constant	3.524^**^	82.263	3.524^**^	82.54	3.517^**^	84.068
Maternal stress	0.337^**^	5.948	0.332^**^	5.88	0.39^**^	6.861
Social support			−0.104^*^	−1.982	−0.125^*^	−2.438
Maternal stress*social support					−0.250^**^	−4.404
Adjust *R^2^*	0.073		0.079		0.117	
*F*-value	35.376		3.928		19.392	

^*^ means *p* < 0.05, ^**^ means *p* < 0.01.

## 4 Discussion

### 4.1 Principal findings

With the rapid advancement of internet technology and the widespread adoption of mobile devices, online health information acquisition has become a crucial approach for pregnant women to meet their healthcare needs. Concurrently, contemporary pregnant women face multifaceted pressures from work, family, and society—these compounding stress factors have further intensified their urgent demand for professional and reliable health information. The research findings will provide empirical evidence for optimizing maternal health information services, holding significant practical implications for enhancing this population’s health literacy.

This study used a questionnaire survey method to collect data through both online and offline channels. The target population consisted of pregnant women, with a final sample of 903 valid responses, including 521 online questionnaires (57.7%) and 382 offline questionnaires (42.3%). For data analysis, the study comprehensively applied mediation and moderation effect models to examine the key factors influencing pregnant women’s online health information-seeking behavior and their underlying mechanisms from multiple dimensions.

According to the study, the quality of online information has a significant positive effect on maternal online health information-seeking behavior. Specifically, when pregnant women perceive online information as authoritative, accurate, and timely, their engagement in online health information searches becomes more frequent. A study on Turkish citizens’ health information-seeking behavior during the COVID-19 pandemic found that trust in official information sources was a key factor influencing their online health information searches (3). Some studies have also revealed that the importance of official source data has increased even more, and reliable and accurate information sources have become critical (44, 45).

The findings indicate that perceived usefulness mediated the relationship between Internet information quality and maternal online health information-seeking behavior. Maternal online health information-seeking behavior was primarily influenced by their perception of the usefulness and reliability of the information. When mothers perceived the online health information as useful with tangible effects, they formed a positive attitude toward Internet browsing for seeking health information, thereby promoting their use of online health information. Our findings are similar to those of Shi et al. (46) and Kim et al. (47), who reported perceived usefulness as a key factor affecting mothers’ willingness to continue using online health information. Overall, perceived usefulness is an important bridge between the quality of online health information and the behavior of online health information (25).

The findings demonstrate a positive correlation between maternal stress levels and online health information-seeking behavior. Starcevic (48) found that individuals with health anxiety are more likely to seek related health information online to alleviate uncertainty. Furthermore, the act of online searching itself may serve as a coping mechanism (34, 49), enabling pregnant women to enhance their sense of control through information acquisition, thereby reducing anxiety. Compared to offline consultations, the Internet provides immediate and low-barrier access to information (50), making it particularly suitable for pregnant women under high-stress conditions.

The research findings revealed that anxiety played a mediating role between maternal stress and online health information-seeking behavior. Stress among professional women comprises work, family, and physiological stressors that directly impact their anxiety (38). As society evolved, mothers assumed multiple roles. Different roles have different demands, which can place different pressures on them. However, role conflict is difficult to resolve, as it can affect their mood and produce anxiety. A study involving Roma communities in Slovakia found that mothers with higher levels of economic insecurity and lower levels of social support felt significantly more stressed (51). In addition, user data privacy is paramount (52). Strengthening data protection for maternal online health information is also an important measure to alleviate their anxiety. The convenience, richness, and anonymity of online health information are favorable for mothers (53, 54).

According to the study, social support played a negative moderating role in the relationship between maternal stress and anxiety. Social support can effectively reduce the impact of anxiety on an individual’s physical and mental health and enhance their ability to withstand pressure. A survey study on older adults’ online health information-seeking behavior found that both social support and professional support significantly enhanced their health awareness, which, in turn, further motivated their efforts to seek relevant health information (55). In addition to the quantity of social support, the quality of social support is crucial. Poor quality of social support can be counterproductive. Considering the differences in the closeness of the relationship between supporters and mothers’ personality characteristics and perceived stress, they may accept different support methods to different degrees (41).

### 4.2 Theoretical and practical implications

Theoretically, this study constructs a dual-path theoretical framework for pregnant women’s online health information-seeking behavior by integrating the Elaboration Likelihood Model and Technology Acceptance Model. It reveals the mediating roles of perceived usefulness and anxiety in the relationship between information quality and seeking behavior, as well as the moderating effect of social support. These findings address the research gap in emotional factors and provide novel theoretical perspectives for health information behavior studies in digital health.

In practice, the research offers concrete guidance for optimizing maternal health information services. Government agencies and health platforms should enhance information quality supervision and source credibility. Medical institutions need to focus on maternal mental health by providing online psychological support. Family members and society should strengthen emotional care to alleviate anxiety. Furthermore, differentiated information services should be designed according to the age and education levels of pregnant women, thereby collectively improving the maternal health information acquisition experience at both the policy and societal levels.

### 4.3 Limitations and future research

Although this study has made contributions at both the theoretical and practical levels, there are still several limitations, providing directions for improvement in future research. At the sample level, the data of this study was gathered from 903 pregnant and postpartum women in China, which may introduce sample selection bias. Future studies can adopt a longitudinal tracking design to expand the sample coverage, such as pregnant and postpartum women from different regions and cultural backgrounds, in order to enhance the universality of the research conclusions. In terms of variable measurement, variables such as stress and anxiety rely on self-rating scales, and there may be social expectation bias. In the future, multimodal measurements can be conducted in combination with physiological indicators (such as cortisol levels) or behavioral data (such as online search records) to enhance the objectivity of the data. Furthermore, the study did not systematically examine the impact of differences in digital literacy among pregnant and postpartum women on information search behavior. In the future, digital literacy can be introduced as a moderating variable to explore its differentiated impact on the effect of information acquisition.

## 5 Conclusion

In the information age, the popularity of the Internet has changed people’s information acquisition behaviors. Based on the “Healthy China 2030” plan and the “three-child” policy of China, this study explored the dynamic mechanism behind online health information-seeking behavior among mothers based on the theory of the ELM and TAM. We proposed a model of the influencing factors of maternal online health information-seeking behavior based on a combination of central and peripheral paths. The results of this study were as follows:

1. Perceived usefulness partially mediated the relationship between Internet information quality and maternal online health information-seeking behavior. In other words, mothers were concerned about both the quality of health information and information sources. The higher the quality of Internet information and information sources, the more the perceived usefulness of online health information among mother, which affects their information-seeking behavior.
2. Anxiety partly mediated the relationship between maternal stress and online health information-seeking behavior. In other words, the greater the perceived stress among mothers, the greater their anxiety, which prompted them to seek online health information for psychological comfort and solutions.
3. Social support had a negative moderating effect on maternal stress and anxiety. Mothers experienced less anxiety when they received greater social support. Therefore, family and friends should provide sufficient care and support to help mothers foster a feeling of safety and reduce their anxiety.

## Data Availability

The original contributions presented in the study are included in the article/supplementary material, further inquiries can be directed to the corresponding author.
